# Supplementary material for: Divergent evolutionary trajectories following speciation in two ectoparasitic honey bee mites
Source: Commun Biol. 2019 Oct 1;2:357. doi: 10.1038/s42003-019-0606-0 (PMC6773775; doi:10.1038/s42003-019-0606-0)
Supplement: Supplementary file 1 — Supplementary Information [file 42003_2019_606_MOESM1_ESM.pdf]

SUPPLEMENTARY FIGURES

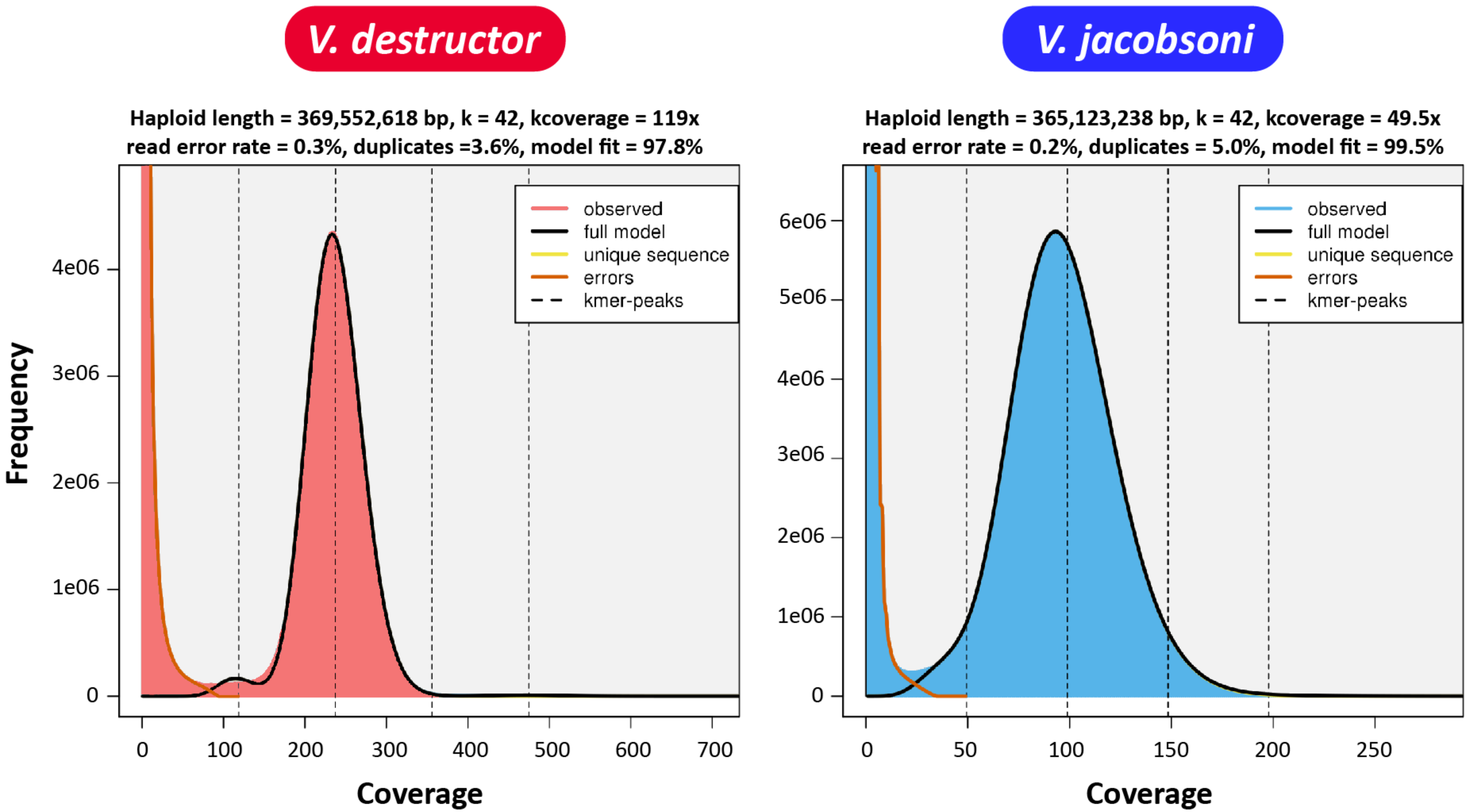
V. destructor
Haploid length = 369,552,618 bp, k = 42, kcoverage = 119x
read error rate = 0.3%, duplicates =3.6%, model fit = 97.8%
observed
full model
unique sequence
errors
kmer-peaks
Frequency
0
1e06
2e06
3e06
4e06
0
100
200
300
400
500
600
700
Coverage
V. jacobsoni
Haploid length = 365,123,238 bp, k = 42, kcoverage = 49.5x
read error rate = 0.2%, duplicates = 5.0%, model fit = 99.5%
observed
full model
unique sequence
errors
kmer-peaks
0
1e06
2e06
3e06
4e06
5e06
6e06
0
50
100
150
200
250
Coverage

Supplementary Figure 1: Similar genome sizes between the two species of Varroa mites estimated from haploid male genome through the k-mer 42 frequency in GenomeScope.

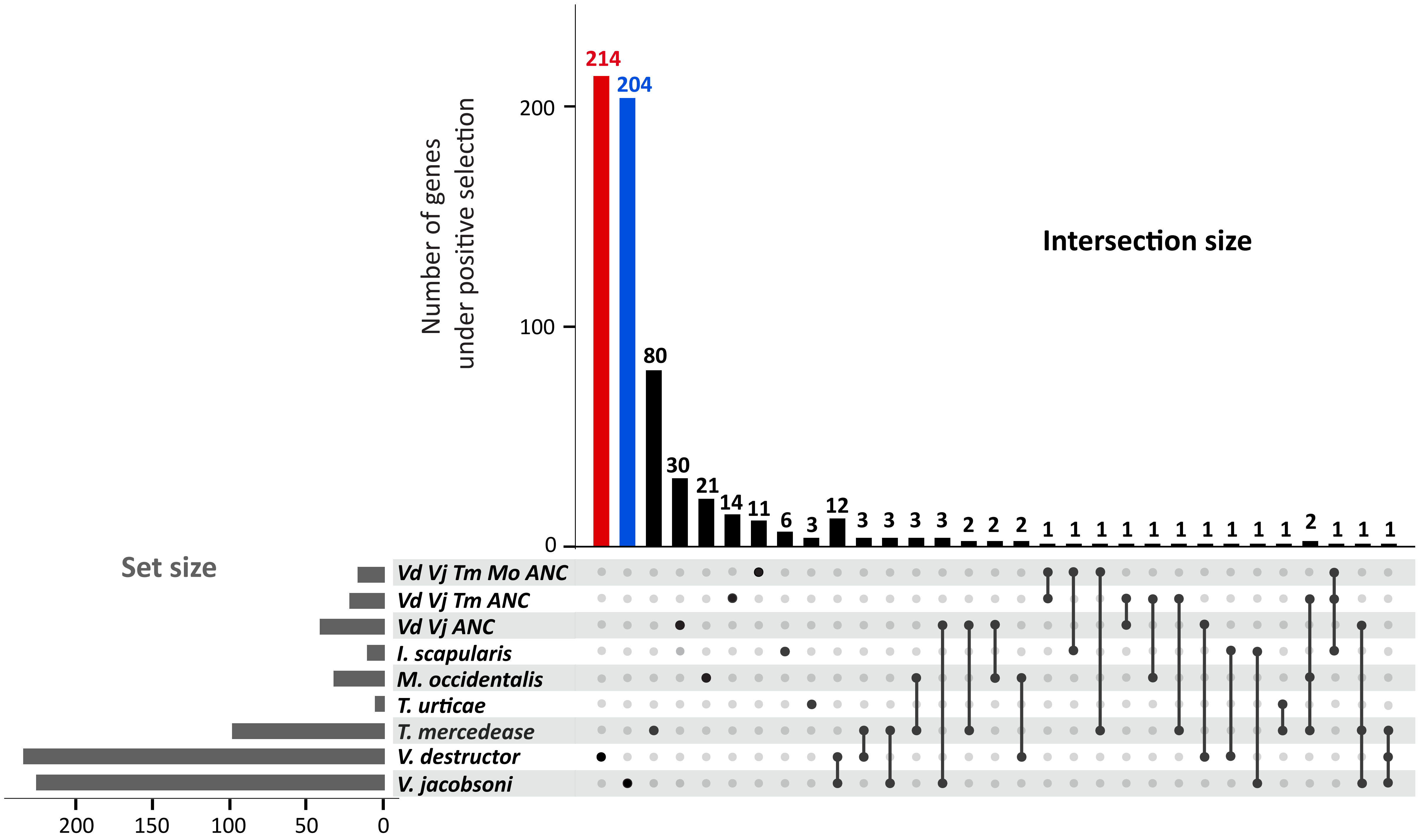

Number of genes under positive selection
Intersection size
214
204
80
30
21
14
11
6
3
12
3
3
3
3
2
2
2
1
1
1
1
1
1
1
1
1
1
2
1
1
1
200
100
0
Set size
Vd Vj Tm Mo ANC
Vd Vj Tm ANC
Vd Vj ANC
I. scapularis
M. occidentalis
T. urticae
T. mercedease
V. destructor
V. jacobsoni
200
150
100
50
0

Supplementary Figure 2: UpSet plot of orthologous genes detected under positive selection detailing the shared number per species. Vd Vj Tm Mo ANC = genes shared with the ancestral and modern Mesostigmata (*V. destructor, V. jacobsoni, T. mercedesae, and M. occidentalis*), Vd Vj Tm ANC = set of genes shared with the ancestor of Asian mites and Vd Vj ANC = set of genes shared with the ancestral Varroidae.

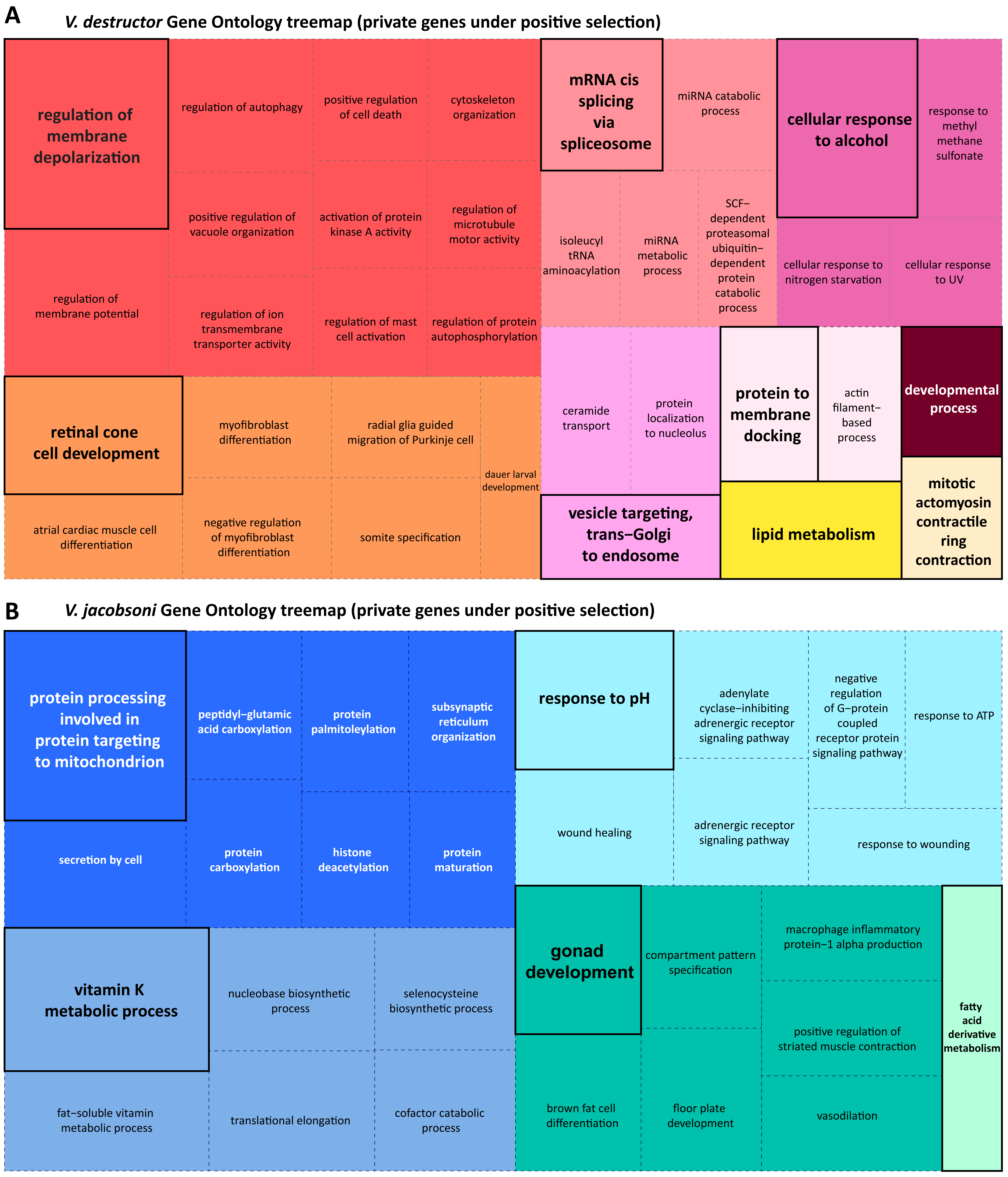
A
V. destructor Gene Ontology treemap (private genes under positive selection)
regulation of membrane depolarization
regulation of autophagy
positive regulation of cell death
cytoskeleton organization
mRNA cis splicing via spliceosome
miRNA catabolic process
cellular response to alcohol
response to methyl methane sulfonate
positive regulation of vacuole organization
activation of protein kinase A activity
regulation of microtubule motor activity
isoleucyl tRNA aminoacylation
miRNA metabolic process
SCF–dependent proteasomal ubiquitin–dependent protein catabolic process
cellular response to nitrogen starvation
cellular response to UV
regulation of membrane potential
regulation of ion transmembrane transporter activity
regulation of mast cell activation
regulation of protein autophosphorylation
ceramide transport
protein localization to nucleolus
protein to membrane docking
actin filament–based process
developmental process
retinal cone cell development
myofibroblast differentiation
radial glia guided migration of Purkinje cell
dauer larval development
atrial cardiac muscle cell differentiation
negative regulation of myofibroblast differentiation
somite specification
vesicle targeting, trans–Golgi to endosome
lipid metabolism
mitotic actomyosin contractile ring contraction
B
V. jacobsoni Gene Ontology treemap (private genes under positive selection)
protein processing involved in protein targeting to mitochondrion
peptidyl–glutamic acid carboxylation
protein palmitoleylation
subsynaptic reticulum organization
response to pH
adenylate cyclase–inhibiting adrenergic receptor signaling pathway
negative regulation of G–protein coupled receptor protein signaling pathway
response to ATP
secretion by cell
protein carboxylation
histone deacetylation
protein maturation
wound healing
adrenergic receptor signaling pathway
response to wounding
vitamin K metabolic process
nucleobase biosynthetic process
selenocysteine biosynthetic process
gonad development
compartment pattern specification
macrophage inflammatory protein–1 alpha production
positive regulation of striated muscle contraction
fatty acid derivative metabolism
fat–soluble vitamin metabolic process
translational elongation
cofactor catabolic process
brown fat cell differentiation
floor plate development
vasodilation

Supplementary Figure 3: Treemap plots (REVIGO) analysis for the summary of GO terms in biological processes for gene detected under positive selection for *V. destructor* (A) and *V. jacobsoni* (B). Related GO terms were joined in clusters (color), and the size of the rectangles was adjusted to reflect the p-value and with a semantic similarity (C = 0.5).

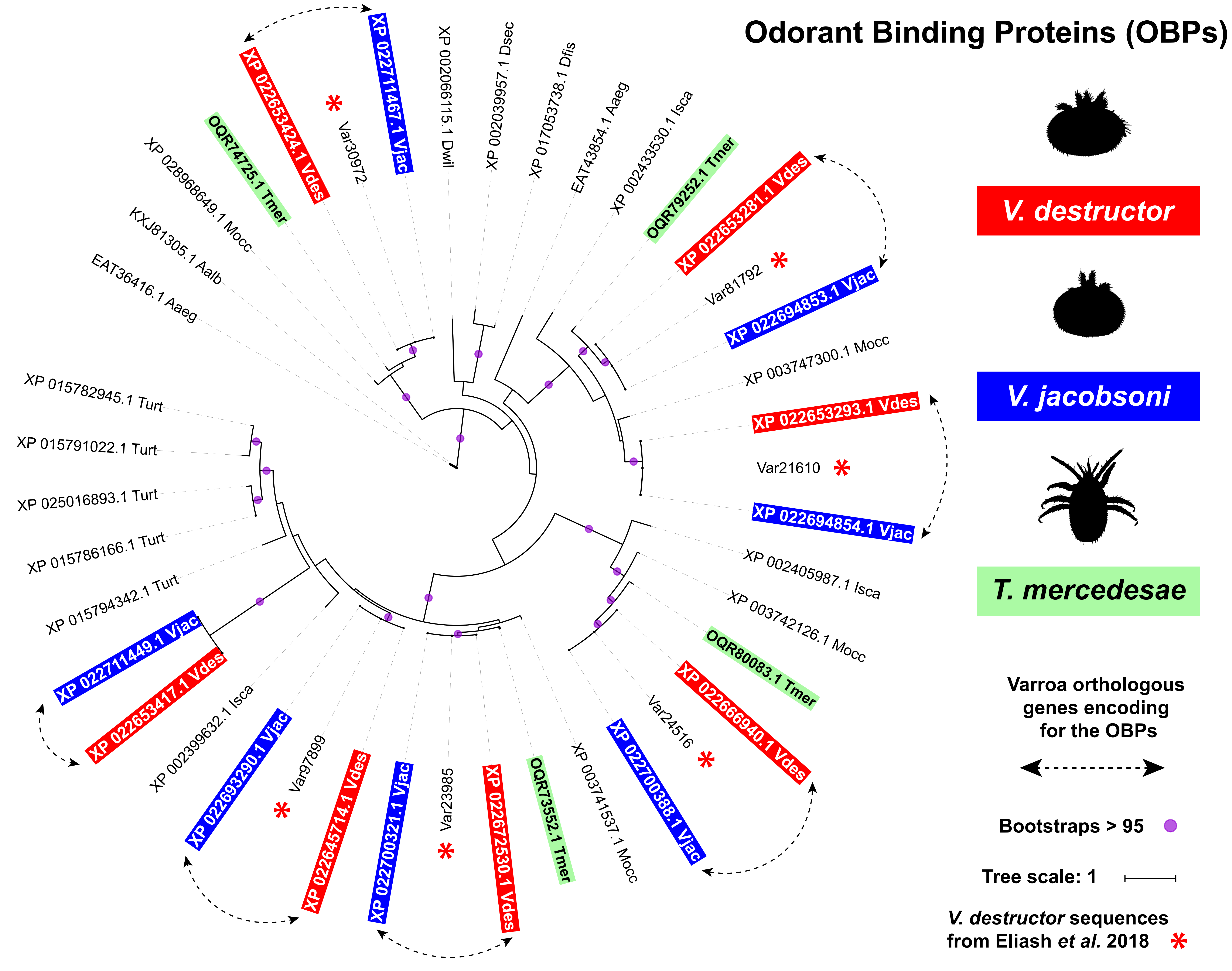

Odorant Binding Proteins (OBPs)
XP 022653424.1 Vdes
XP 022711467.1 Vjac
Var30972
OQR74725.1 Tmer
XP 002066115.1 Dwil
XP 002039957.1 Dsec
XP 017053738.1 Dfis
EAT43854.1 Aaeg
XP 002433530.1 Isca
OQR79252.1 Tmer
XP 022653281.1 Vdes
Var81792
XP 022694853.1 Vjac
XP 003747300.1 Mocc
XP 022653293.1 Vdes
Var21610
XP 022694854.1 Vjac
XP 002405987.1 Isca
XP 003742126.1 Mocc
OQR80083.1 Tmer
XP 022666940.1 Vdes
Var24516
XP 022700388.1 Vjac
XP 003741537.1 Mocc
OQR73552.1 Tmer
XP 022672530.1 Vdes
Var23985
XP 022700321.1 Vjac
XP 022645714.1 Vdes
Var97899
XP 022693290.1 Vjac
XP 002399632.1 Isca
XP 022653417.1 Vdes
XP 022711449.1 Vjac
XP 015794342.1 Turt
XP 015786166.1 Turt
XP 025016893.1 Turt
XP 015791022.1 Turt
XP 015782945.1 Turt
EAT36416.1 Aaeg
KXJ81305.1 Aalb
XP 028968649.1 Mocc
V. destructor
V. jacobsoni
T. mercedesae
Varroa orthologous genes encoding for the OBPs
Bootstraps > 95
Tree scale: 1
V. destructor sequences from Eliash et al. 2018

Supplementary Figure 4: OBP repertoires are highly conserved between Varroa sibling species and other Acari. The phylogenetic tree was constructed using 42 amino acid sequences from Acari. *V. destructor* (Vdes = genome and Var = protein sequences from previous chemosensory study [1]), *V. jacobsoni* (Vjac), *T. mercedesae* (Tmer), *M. occidentalis* (Mocc), *I. scapularis* (Isca), *T. urticae* (Turt) (see the list in Supplementary Table 9). Additionally, sequences from mosquitos *Aedes aegypti* (Aaeg), *A. albopictus* (Aaeg) and Drosophila fruit flies (D), were downloaded from NCBI accession and aligned with MAFFT. Best fit model computed for the tree is WAG+I+G4 using IQ-TREE. Bootstrap values were estimated using an SH-like aLRT with 1000 and bootstraps over 95% are shown by a purple circle.

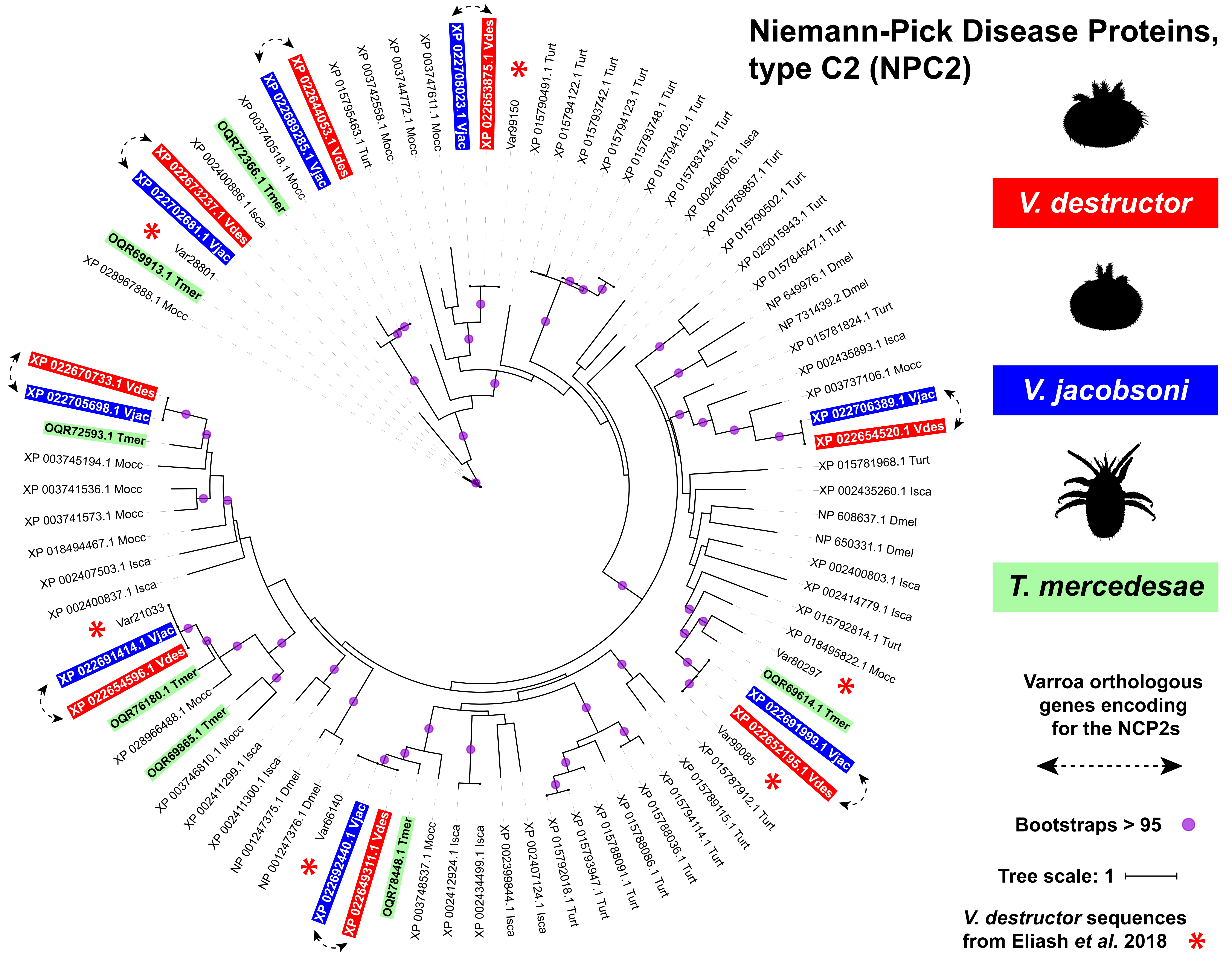

Niemann-Pick Disease Proteins, type C2 (NPC2)
V. destructor
V. jacobsoni
T. mercedesae
Varroa orthologous genes encoding for the NCP2s
Bootstraps > 95
Tree scale: 1
V. destructor sequences from Eliash et al. 2018
XP 022653875.1 Vdes
XP 022708023.1 Vjac
XP 003747611.1 Mocc
XP 003744772.1 Mocc
XP 003742558.1 Mocc
XP 015795463.1 Turt
XP 022644053.1 Vdes
XP 022689285.1 Vjac
XP 003740518.1 Mocc
OQR72366.1 Tmer
XP 002400886.1 Isca
XP 022673237.1 Vdes
XP 022702681.1 Vjac
Var28801
OQR69913.1 Tmer
XP 028967888.1 Mocc
Var99150
XP 015790491.1 Turt
XP 015794122.1 Turt
XP 015793742.1 Turt
XP 015794123.1 Turt
XP 015793748.1 Turt
XP 015794120.1 Turt
XP 015793743.1 Turt
XP 002408676.1 Isca
XP 015789857.1 Turt
XP 015790502.1 Turt
XP 025015943.1 Turt
XP 015784647.1 Turt
NP 649976.1 Dmel
NP 731439.2 Dmel
XP 015781824.1 Turt
XP 002435893.1 Isca
XP 003737106.1 Mocc
XP 022706389.1 Vjac
XP 022654520.1 Vdes
XP 015781968.1 Turt
XP 002435260.1 Isca
NP 608637.1 Dmel
NP 650331.1 Dmel
XP 002400803.1 Isca
XP 002414779.1 Isca
XP 015792814.1 Turt
XP 018495822.1 Mocc
Var80297
OQR69614.1 Tmer
XP 022691999.1 Vjac
XP 022652195.1 Vdes
Var99085
XP 015787912.1 Turt
XP 015789115.1 Turt
XP 015794114.1 Turt
XP 015788036.1 Turt
XP 015788086.1 Turt
XP 015788091.1 Turt
XP 015793947.1 Turt
XP 015792018.1 Turt
XP 002407124.1 Isca
XP 002399844.1 Isca
XP 002434499.1 Isca
XP 002412924.1 Isca
XP 003748537.1 Mocc
OQR78448.1 Tmer
XP 022649311.1 Vdes
XP 022692440.1 Vjac
Var66140
NP 001247376.1 Dmel
NP 001247375.1 Dmel
XP 002411300.1 Isca
XP 002411299.1 Isca
XP 003746810.1 Mocc
OQR69865.1 Tmer
XP 028966488.1 Mocc
OQR76180.1 Tmer
XP 022654596.1 Vdes
XP 022691414.1 Vjac
Var21033
XP 002400837.1 Isca
XP 002407503.1 Isca
XP 018494467.1 Mocc
XP 003741573.1 Mocc
XP 003741536.1 Mocc
XP 003745194.1 Mocc
OQR72593.1 Tmer
XP 022705698.1 Vjac
XP 022670733.1 Vdes

Supplementary Figure 5: *V. destructor* and *V. jacobsoni* genomes showed eight NCP2 amino acid sequences mostly conserved with the other honey bee parasite *T. mercedesae*. The phylogenetic tree was constructed using 86 amino acid sequences from Acari *V. destructor* (Vdes = genome and Var = protein sequences from previous chemosensory study [1]), *V. jacobsoni* (Vjac), *T. mercedesae* (Tmer), *M. occidentalis* (Mocc), *I. scapularis* (Isca) and *T. urticae* (Turt) (see the list in Supplementary Table 9). Additionally, sequences from the fruit fly *Drosophila melanogaster* (Dmel), downloaded from NCBI and aligned with MAFFT. Best fit model computed for the tree is LG+R4 using IQ-TREE. Bootstrap values were estimated using an SH-like aLRT with 1000 and bootstraps over 95% are shown by a purple circle.

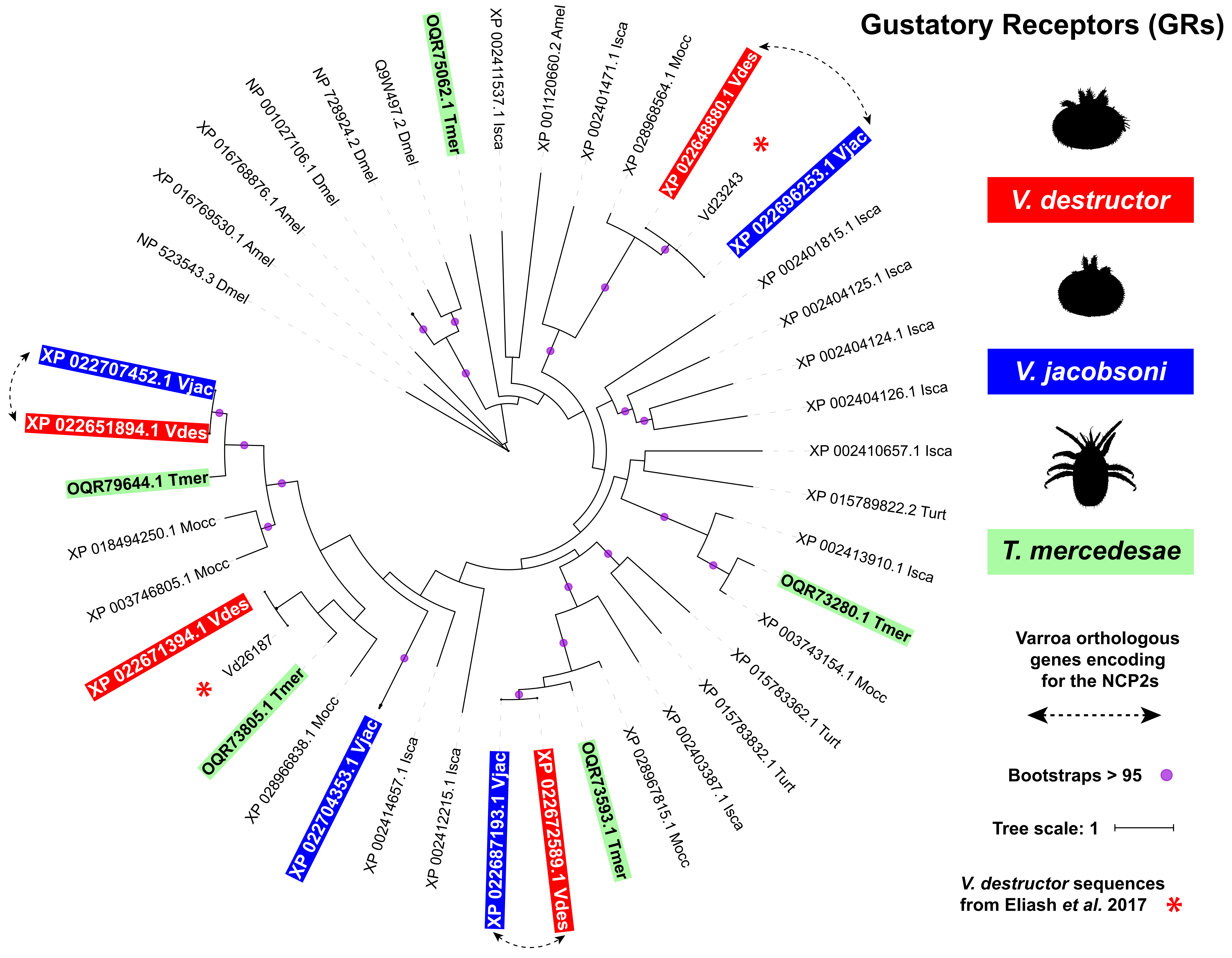

Gustatory Receptors (GRs)
V. destructor
V. jacobsoni
T. mercedesae
Varroa orthologous genes encoding for the NCP2s
Bootstraps > 95
Tree scale: 1
V. destructor sequences from Eliash et al. 2017
XP 022648880.1 Vdes
Vd23243
XP 022696253.1 Vjac
XP 028968564.1 Mocc
XP 002401471.1 Isca
XP 001120660.2 Amel
XP 002411537.1 Isca
OQR75062.1 Tmer
Q9W497.2 Dmel
NP 728924.2 Dmel
NP 001027106.1 Dmel
XP 016768876.1 Amel
XP 016769530.1 Amel
NP 523543.3 Dmel
XP 022707452.1 Vjac
XP 022651894.1 Vdes
OQR79644.1 Tmer
XP 018494250.1 Mocc
XP 003746805.1 Mocc
XP 022671394.1 Vdes
Vd26187
OQR73805.1 Tmer
XP 028966838.1 Mocc
XP 022704353.1 Vjac
XP 002414657.1 Isca
XP 002412215.1 Isca
XP 022687193.1 Vjac
XP 022672589.1 Vdes
OQR73593.1 Tmer
XP 028967815.1 Mocc
XP 002403387.1 Isca
XP 015783832.1 Turt
XP 015783362.1 Turt
XP 003743154.1 Mocc
OQR73280.1 Tmer
XP 002413910.1 Isca
XP 015789822.2 Turt
XP 002410657.1 Isca
XP 002404126.1 Isca
XP 002404124.1 Isca
XP 002404125.1 Isca
XP 002401815.1 Isca

Supplementary Figure 6: *V. destructor* and *V. jacobsoni* genomes presented only four gustatory receptors amino acid sequences-like. The phylogenetic tree was constructed using 45 amino acid sequences from Acari *V. destructor* (Vdes = genome and Var = protein sequences from previous chemosensory study [1]), *V. jacobsoni* (Vjac), *T. mercedesae* (Tmer), *M. occidentalis* (Mocc), *I. scapularis* (Isca) and *T. urticae* (Turt) (see the list in Supplementary Table 9). Additionally, sequences from the fruit fly *Drosophila melanogaster* (Dmel) and the honey bee *A. mellifera* (Amel), sequences were downloaded from NCBI and aligned with MAFFT. The best-fit model computed for the tree is LG+F+R4 using IQ-TREE. Bootstrap values were estimated using an SH-like aLRT with 1000 and bootstraps over 95% are shown by a purple circle.

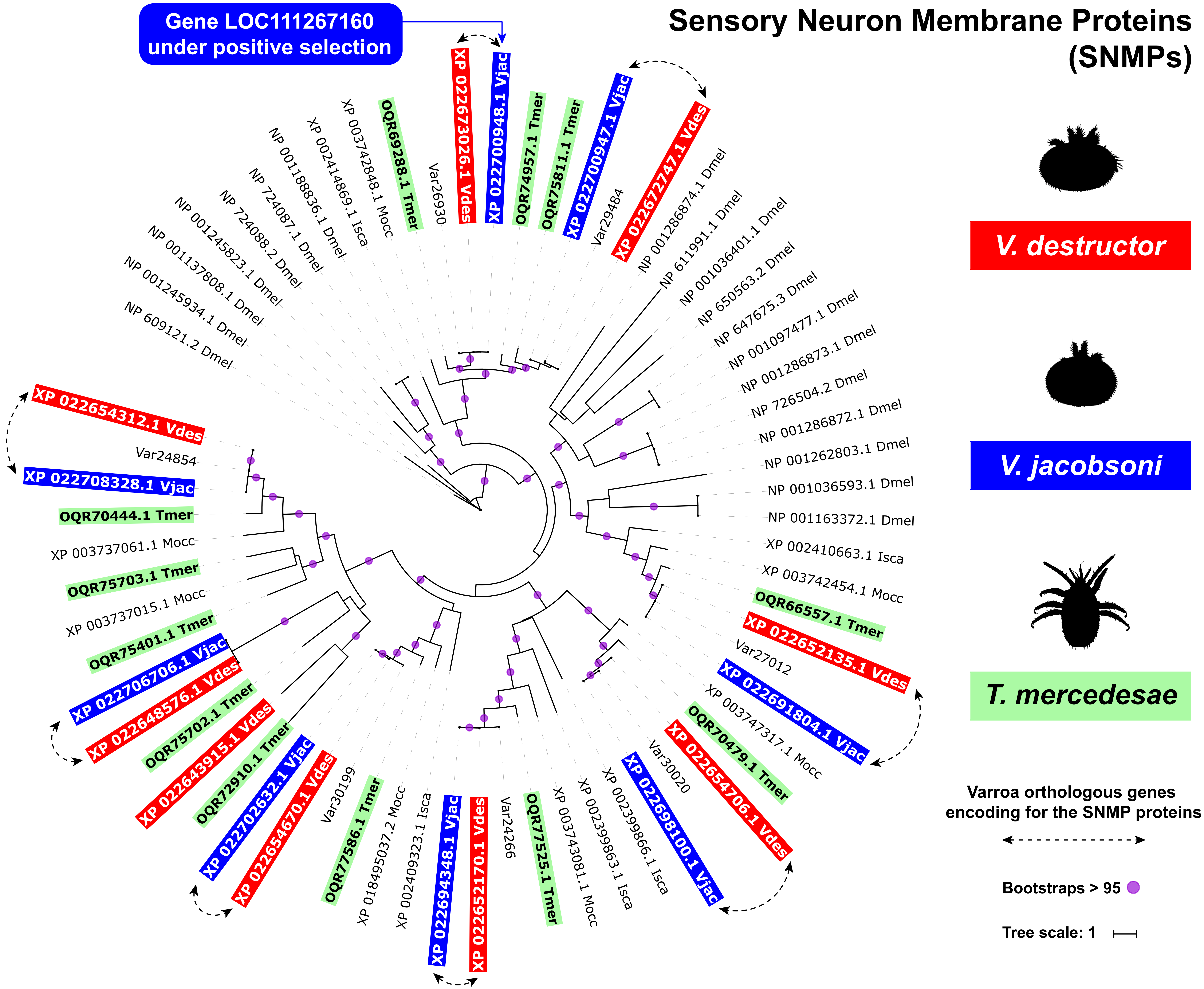

Gene LOC111267160 under positive selection
Sensory Neuron Membrane Proteins (SNMPs)
V. destructor
V. jacobsoni
T. mercedesae
Varroa orthologous genes encoding for the SNMP proteins
Bootstraps > 95
Tree scale: 1
XP 022673026.1 Vdes
XP 022700948.1 Vjac
OQR74957.1 Tmer
OQR75811.1 Tmer
XP 022700947.1 Vjac
Var29484
XP 022672747.1 Vdes
NP 001286874.1 Dmel
NP 611991.1 Dmel
NP 001036401.1 Dmel
NP 650563.2 Dmel
NP 647675.3 Dmel
NP 001097477.1 Dmel
NP 001286873.1 Dmel
NP 726504.2 Dmel
NP 001286872.1 Dmel
NP 001262803.1 Dmel
NP 001036593.1 Dmel
NP 001163372.1 Dmel
XP 002410663.1 Isca
XP 003742454.1 Mocc
OQR66557.1 Tmer
XP 022652135.1 Vdes
Var27012
XP 022691804.1 Vjac
XP 003747317.1 Mocc
OQR70479.1 Tmer
XP 022654706.1 Vdes
Var30020
XP 022698100.1 Vjac
XP 002399866.1 Isca
XP 002399863.1 Isca
XP 003743081.1 Mocc
OQR77525.1 Tmer
Var24266
XP 022652170.1 Vdes
XP 022694348.1 Vjac
XP 002409323.1 Isca
XP 018495037.2 Mocc
OQR77586.1 Tmer
Var30199
XP 022654670.1 Vdes
XP 022702632.1 Vjac
OQR72910.1 Tmer
XP 022643915.1 Vdes
OQR75702.1 Tmer
XP 022648576.1 Vdes
XP 022706706.1 Vjac
OQR75401.1 Tmer
XP 003737015.1 Mocc
OQR75703.1 Tmer
XP 003737061.1 Mocc
OQR70444.1 Tmer
XP 022708328.1 Vjac
Var24854
XP 022654312.1 Vdes
NP 609121.2 Dmel
NP 001245934.1 Dmel
NP 001137808.1 Dmel
NP 001245823.1 Dmel
NP 724088.2 Dmel
NP 724087.1 Dmel
NP 001188836.1 Dmel
XP 002414869.1 Isca
XP 003742848.1 Mocc
OQR69288.1 Tmer
Var26930

Supplementary Figure 7: Sensory neuron membrane protein repertoires are conserved among honey bee parasites, but sibling Varroa species show signs of differential selective regimes. The phylogenetic tree was constructed using 67 amino acid sequences from Acari *V. destructor* (Vdes = genome and Var = protein sequences from previous chemosensory study [1]), *V. jacobsoni* (Vjac), *T. mercedesae* (Tmer), *M. occidentalis* (Mocc), *I. scapularis* (Isca) (see list in Supplementary Table 9). Additionally, sequences from the fruit fly *Drosophila melanogaster* (Dmel), were implemented with NCBI accession and aligned with MAFFT. Best fit model computed for the tree is LG+R5 using IQ-TREE. Bootstrap values were estimated using an SH-like aLRT with 1000 and bootstraps over 95% are shown by a purple circle.
