# Supplementary material for: Divergent evolutionary trajectories following speciation in two ectoparasitic honey bee mites
Source: Commun Biol. 2019 Oct 1;2:357. doi: 10.1038/s42003-019-0606-0 (PMC6773775; doi:10.1038/s42003-019-0606-0)
Supplement: Supplementary file 2 — Description of Additional Supplementary Files [file 42003_2019_606_MOESM2_ESM.docx]

**Description of Additional Supplementary Files**

**File Name**: Supplementary Data 1

**Description**:  Supplementary Table in excel format, with the summary output of detected ortholog genes and correspondence among the six species included in the phylogeny from ORTHONOME pipeline. SOG = super orthologue group, OG = orthologue group.

**File Name**: Supplementary Data 2

**Description**:  Supplementary Table in excel format, with the GO terms enriched from ortholog clusters before species split between Varroa mites and with four other Acari. Lineage = phylogenetic delimitation for orthologues groups (ROOT = 6 species, PARASITIFORMES = 5 species, MESOSTIGMATA = 4 species, ASIAN-MITES = *T. mercedesae*, *V. destructor* and *V. jacobsoni*) and VARROIDAE (Varroa mites only).

**File Name**: Supplementary Data 3

**Description**:  Supplementary Table in excel format, with the genomic details of the genes detected under positive either on *V. destructor, V. jacobsoni* or Varroa ancestral (before split) branch. gene.no = gene ID code, gene.id = full gene ID, GenBank.accession = localization in *V. destructor* chromosome.

**File Name**: Supplementary Data 4

**Description**:  Supplementary Table in excel format, with the details of GO terms enrichment analysis of genes detected under positive selection for *V. destructor, V. jacobsoni* and Varroa ancestral branch (before species split). GO = Gene Ontology, BP = Biology Process.

**File Name**: Supplementary Data 5

**Description**:  Supplementary Table in excel format, with the list of genes duplicated in tandem different between *V. destructor* and *V. jacobsoni* from the MSOAR2 pipeline. In red font, gene removed from duplication events as potential assembly artifact.

**File Name**: Supplementary Data 6

**Description**:  Supplementary Table in excel format, with the list of genes duplicated in tandem shared by *Varroa* and *Tropilaelaps* mites.

**File Name**: Supplementary Data 7

**Description**:  Supplementary Table in excel format, with the details of GO terms enrichment analysis of duplicated in tandem genes for *V. destructor* and *V. jacobsoni*. GO = Gene Ontology, BP = Biology Process.

**File Name**: Supplementary Data 8

**Description**:  Supplementary Table in excel format, with the list of gene sets enrichment analysis of genes duplicated shared by Varroa mites. GO = Gene Ontology, BP = Biology Process.

**File Name**: Supplementary Data 9

**Description**:  Supplementary Table in excel format, with the details of the proteins and related genes used for phylogenetic analysis and comparison of chemosensory proteins. OBP = Odorant Binding Protein, NCP2 = Niemann-Pick Disease Protein, type2, SNMP = Sensory Neuron Membrane Protein, GR = Glutamate Receptor, and IGR = Ionotropic Glutamate Receptor.
