# Supplementary material for: Divergent evolutionary trajectories following speciation in two ectoparasitic honey bee mites
Source: Commun Biol. 2019 Oct 1;2:357. doi: 10.1038/s42003-019-0606-0 (PMC6773775; doi:10.1038/s42003-019-0606-0)
Supplement: Supplementary file 12 — Reporting Summary [file 42003_2019_606_MOESM12_ESM.pdf]

nature research

Corresponding author(s): Maeva A. Techer and Alexander S. Mikheyev

Last updated by author(s): Sep 10, 2019

Reporting Summary

Nature Research wishes to improve the reproducibility of the work that we publish. This form provides structure for consistency and transparency in reporting. For further information on Nature Research policies, see Authors & Referees and the Editorial Policy Checklist.

Statistics

For all statistical analyses, confirm that the following items are present in the figure legend, table legend, main text, or Methods section.

n/a	Confirmed	
☐	☒	The exact sample size (*n*) for each experimental group/condition, given as a discrete number and unit of measurement
☐	☒	A statement on whether measurements were taken from distinct samples or whether the same sample was measured repeatedly
☐	☒	The statistical test(s) used AND whether they are one- or two-sided<br>*Only common tests should be described solely by name; describe more complex techniques in the Methods section.*
☒	☐	A description of all covariates tested
☐	☒	A description of any assumptions or corrections, such as tests of normality and adjustment for multiple comparisons
☐	☒	A full description of the statistical parameters including central tendency (e.g. means) or other basic estimates (e.g. regression coefficient) AND variation (e.g. standard deviation) or associated estimates of uncertainty (e.g. confidence intervals)
☐	☒	For null hypothesis testing, the test statistic (e.g. *F*, *t*, *r*) with confidence intervals, effect sizes, degrees of freedom and *P* value noted<br>*Give P values as exact values whenever suitable.*
☒	☐	For Bayesian analysis, information on the choice of priors and Markov chain Monte Carlo settings
☒	☐	For hierarchical and complex designs, identification of the appropriate level for tests and full reporting of outcomes
☒	☐	Estimates of effect sizes (e.g. Cohen's *d*, Pearson's *r*), indicating how they were calculated

Our web collection on statistics for biologists contains articles on many of the points above.

Software and code

Policy information about availability of computer code

Data collection	The sequences data used for the assembly were produced for this study from Varroa mites from Okinawa in Japan and Papua New Guinea at the Okinawa Institute of Science and Technology. The four other Acari genomes used were downloaded from Genbank. The map distribution of Varroa mites on their original host was build from reviewing the litterature and each geopoint are available on our GitHub account. Finally the map was produced using QGIS 2.16.1 (Open Source Geospatial Foundation Project http://qgis.osgeo.org) and the public domain natural 10m earth raster NE1_HR_LC (https://github.com/nvkelso/natural-earth-raster).
Data analysis	Open access software used for genomic assembly and analyses: ParDre, DeConSeq, PEAR, Newbler (v. 2.6), Trinity, BWA-MEM. jellyfish v. 2.2.7, GenomeScope, PBJelly, NCBI eukaryotic annotation pipeline, Orthonome, R (UpSet plots, GOstats, ggplot2), , HyPhy, REVIGO, NextGenMap 0.5.0, Samtools 1.3.1, VariantBam 1.4.3, FreeBayes v1.1.0-46, Vcflib, ClustalW, Mega 7.0.18. All softwares used were indicated in the manuscript with the command lines if parameters were not chosen by default. The R code is available on an online resource github.com/MikheyevLab/varroa-denovo-genomes.

For manuscripts utilizing custom algorithms or software that are central to the research but not yet described in published literature, software must be made available to editors/reviewers. We strongly encourage code deposition in a community repository (e.g. GitHub). See the Nature Research guidelines for submitting code & software for further information.

Data

Policy information about availability of data

All manuscripts must include a data availability statement. This statement should provide the following information, where applicable:

- Accession codes, unique identifiers, or web links for publicly available datasets
- A list of figures that have associated raw data
- A description of any restrictions on data availability

All data generated or analysed during this study are included in this published article (and its supplementary information files). V. destructor (Vdes_3.0) and V. jacobsoni (Vjacob_1.0) assembled reference genomes and annotation are available on NCBI database respectively with the accession number GCF_002443255 and GCF_002532875. Raw Illumina and PacBio reads are also available in the Sequence Read Archive (SRA) under the Bioprojects PRJDB6279 and PRJNA391052. Online

supplemental codes and map are available with an R markdown hosted from on github.com/MaevaTecherMikheyevLab/varroa-denovo-genomes. Raw data of both Varroa mites micro-CT scans are available upon request.

Field-specific reporting

Please select the one below that is the best fit for your research. If you are not sure, read the appropriate sections before making your selection.

☐ Life sciences ☐ Behavioural & social sciences ☒ Ecological, evolutionary & environmental sciences

For a reference copy of the document with all sections, see nature.com/documents/nr-reporting-summary-flat.pdf

Ecological, evolutionary & environmental sciences study design

All studies must disclose on these points even when the disclosure is negative.

Study description

Assembly de novo of the two sister species Varroa destructor and Varroa jacobsoni, specialist ectoparasites of Apis honey bee. Comparative genomic analyses was used between the two mites to test whether the species may may pursue similar or different strategies of adaptation to the same host.

Research sample

Varroa destructor and Varroa jacobsoni (Acari, Mesostigmata) are cryptic sibling species both originally parasiting the Eastern honey bee (Apis cerana) and switch host to the Western honey bee (Apis mellifera). Following the host switch about 100 years, V. destructor globally spread worldwide and is now considered as the main driver of honey bee colony losses. V. jacobsoni is for now restricted to South-Asia - Indonesian region and jumped on A. mellifera about 10 years ago in Papua New Guinea. All samples of V. destructor were collected on A. mellifera colonies from OIST experimental apiary in Okinawa, Japan (26.477 N 127.835 E) in August-September 2016. All samples of V. jacobsoni were collected form the first detection survey in 2008 in Papua New Guinea on A. mellifera.

Sampling strategy

One mature male V. destructor was collected within a worker cell at the red-eye pupa developmental stage using a brush and kept in absolute ethanol at -80C. Mature adult females were collected from adult honey bee workers. In order to obtain a large number of mites infecting adult workers, we modified the standard powdered sugar method for an entire hive as follows: one hive super was placed on top of a white collecting tray, containing three to four frames with honey bee workers and no queen; approximately 500g of powdered sugar was then applied using sieve filter powder strainer; the sugar and honey bees were shaken in the tray for 2 min and then separated using a sieve filter, placing the sugared-workers directly within hive. This process was repeated on adjacent colonies. Varroa mites were separated from their hosts and trapped in the icing sugar. Following a water rinse on a gauze mesh, a 170 total of 1,207 alive Varroa females were snap frozen at -80C until laboratory processing (inactive, sluggish or dead mites were discarded).
One mature male V. jacobsoni (VJ856) collected from A. mellifera drone brood cell in Papua New Guinea (between EHP and Henganofi border, 30/05/2008) was used to prepare whole genome library.

Data collection

In addition to the genomic sequences we generated, we used available reference genomes available NCBI for Tropilaelaps mercedesae, Ixodes scapularis, Metaseiulus occidentalis and Tetranychus urticae to carry out positive selection analysis. We also downloaded from NCBI V. destructor mitogenome (NC_004454.2) along with 26 sequences of partial Cox1 mtDNA gene and 22 concatenated sequences of Cox1,Cox3, Atp6 and cytB of Varroa spp. to conduct phylogenetic analyses.

Timing and spatial scale

Punctual sampling

Data exclusions

No data were excluded.

Reproducibility

Genomes assembly and raw sequences are freely available from DDBJ or GenBank database to reproduce using our computational method described. Prediction of orthologs was computed using the ORTHONOME pipeline and additional four Acari genomes for which accession numbers are given in the manuscritp. The online resource for R code is in markdown, allowing anyone to reproduce the statistical analysis on gene selection overlaps.

Randomization

N/A

Blinding

N/A

Did the study involve field work? ☒ Yes ☐ No

Field work, collection and transport

Field conditions

The field work specifically conducted for collecting enough DNA material was done in OIST apiary from August-September 2016. At this time, it is summer with little rainfall. This is also a period in which Varroa mites populations increase rapidly with the availability of drone and worker brood in A. mellifera colonies. For V. jacobsoni samples, the sampling conditions are detailed in Roberts et al. 2015 (Mol. Ecol)

Location

Okinawa, Japan (V. destructor) and Papua New Guinea (V. jacobsoni)

Access and import/export

All A. mellifera colonies naturally infested by V. destructor at OIST experimental apiary and related activities were declared to the

Access and import/export	Okinawa governor. Collaboration between CSIRO and Okinawa Institute of Science and Technology via the Ecology and Evolution lab was establish to process in laboratory V. jacobsoni samples.
Disturbance	We used icing sugar on A. mellifera colonies to collect V. destructor at OIST in order to disturb the less possible the colonies. Contrary to more invasive and destructive method like ethanol wash, powdered icing sugar allows to collect mites without harming unnecessarily honey bee hosts.

Reporting for specific materials, systems and methods

We require information from authors about some types of materials, experimental systems and methods used in many studies. Here, indicate whether each material, system or method listed is relevant to your study. If you are not sure if a list item applies to your research, read the appropriate section before selecting a response.

Materials & experimental systems

n/a	Involved in the study
☒	☐ Antibodies
☒	☐ Eukaryotic cell lines
☒	☐ Palaeontology
☐	☒ Animals and other organisms
☒	☐ Human research participants
☒	☐ Clinical data

Methods

n/a	Involved in the study
☒	☐ ChIP-seq
☒	☐ Flow cytometry
☒	☐ MRI-based neuroimaging

Animals and other organisms

Policy information about studies involving animals; ARRIVE guidelines recommended for reporting animal research

Laboratory animals	No laboratory animals were involved in this study.
Wild animals	Varroa destructor and Varroa jacobsoni.
Field-collected samples	Varroa destructor (males and female) and Varroa jacobsoni (males and female) parasites
Ethics oversight	Varroa mites and more particularly V. destructor are listed in the global invasive species database (http://www.iucngisd.org) and no ethical approval was required. Regarding the manipulation of their honey bee host upon collection. A. mellifera is an invertebarte and no specific ethic approval was required.

Note that full information on the approval of the study protocol must also be provided in the manuscript.
